# Prognostic value of the serum apolipoprotein B to apolipoprotein A-I ratio in metastatic colorectal cancer patients

**DOI:** 10.7150/jca.35659

**Published:** 2020-01-01

**Authors:** Dong-Dong Yang, Zhan-Hong Chen, De-Shen Wang, Hong-En Yu, Jia-Huan Lu, Rui-Hua Xu, Zhao-Lei Zeng

**Affiliations:** 1 Sun Yat-sen University Cancer Center, State Key Laboratory of Oncology in South China, Collaborative Innovation Center for Cancer Medicine, 651 Dong fengdong Road, Guangzhou, 510060, China.; 2 Department of Medical Oncology and Guangdong Key Laboratory of Liver Disease, the Third Affiliated Hospital of Sun Yat-sen University, Guangzhou, China.

**Keywords:** ApoB to ApoA-I ratio, metastatic colorectal cancer, overall survival, prognostic factor, retrospective study.

## Abstract

**Background:** The aim of our research was to assess the prognostic value of the apolipoprotein B (ApoB) to apolipoprotein A-I (ApoA-I) ratio (ApoB/ApoA-I) in metastatic colorectal cancer (mCRC) patients.

**Methods:** We randomly assigned 838 patients into the training cohort (n=578) and the validation cohort (n=260). The cut-off value of the ApoB/ApoA-I in the training cohort identified by a receiver operating characteristic (ROC) curve was 0.69 and was further validated in the validation cohort. A propensity score matching (PSM) analysis was carried out to eliminate the imbalance in the baseline characteristics of the high and low ApoB/ApoA-I group. The PSM cohort of 542 mCRC patients was generated. We also validated our main findings and conclusions with an independent cohort (n=150). Univariate and multivariate analyses were conducted to explore the independent prognostic value of the ApoB/ApoA-I in the training cohort (n=578), the validation cohort (n=260), the PSM cohort (n=542) and the independent cohort (n=150).

**Results:** Patients in the high ApoB/ApoA-I group had significantly shorter overall survival compared to those in the low ApoB/ApoA-I group in the training cohort, the validation cohort, the PSM cohort and the independent cohort (*P* <0.01). Multivariate analysis indicated that the ApoB/ApoA-I was an independent prognostic index for OS in the training cohort [hazard ratio (HR):1.371; 95% confidence interval (CI):1.205-1.870, *P*=0.045], the validation cohort (HR: 1.924; 95% CI: 1.360-2.723, *P*<0.001), the PSM cohort (HR: 1.599; 95% CI: 1.287-1.988,* P*<0.001) and the independent cohort (HR: 1.949; 95% CI: 1.014-3.747,* P*=0.046).

**Conclusions:** An increased baseline serum ApoB/ApoA-I is an independent prognostic factor for a poor prognosis in mCRC patients.

## Introduction

Colorectal cancer (CRC) was the fourth most common malignant cancer and the second leading cause of cancer-related deaths worldwide in 2018. More than 20% of CRC patients were diagnosed with distant metastasis at initial diagnosis. Although obvious progress was achieved in the fields of molecular targeted therapy, immunotherapy and multidisciplinary treatment (MDT), the 5-year overall survival (OS) rate of metastatic colorectal cancer (mCRC) is still below 20% 1-3. Different mCRC patients may have different prognoses, with OS varying from several months to several years. Factors such as liver oligometastasis, RAS mutation and BRAF mutation status have been reported to be prognostic of OS and can also guide treatment options for mCRC patients. The identification of new independent prognostic factors for mCRC is important and will help us better predict the prognosis of mCRC patients. Lipid disequilibrium has been reported to contribute to the occurrence and development of CRC. Clinical parameters reflecting lipid disequilibrium may serve as prognostic factors for mCRC patients. An increased low-density lipoprotein cholesterol (LDL-C) to high-density lipoprotein cholesterol (HDL-C) ratio (LHR) has been reported to predict a poor prognosis for mCRC patients with elevations in LDL-C 4-6. However, little is known about the prognostic value of the ApoB to ApoA-I ratio (ApoB/ApoA-I) in mCRC patients.

Apolipoproteins have been reported to be extensively involved in the processes of cancer occurrence, progression and treatment 7-10. Several studies have suggested that apolipoproteins can participate in carcinogenesis by promoting proliferation and invasion, enhanced antitumor immunity or drug delivery, and an immediate oxidative stress reaction^11^-^16^. Apolipoprotein A-I (ApoA-I), mainly synthesized in the liver and the small intestine, is a major fraction of HDL-C, accounting for approximately 70% 17, 18. In CRC patients, the serum ApoA-I level has been reported to be an independent prognostic indicator to predict chemotherapy efficacy and survival time 19-23. On the other hand, apolipoprotein B (ApoB), an important blood apolipoprotein existing mostly in LDL-C and very-low-density lipoprotein cholesterol (VLDL-C), has been paid less attention to in CRC than ApoA-I. Päivi Sirniö et al. analyzed the correlation between the serum ApoB/ApoA-I and survival in 144 CRC patients. They found that a decreased ApoB/ApoA-I was associated with improved cancer-specific survival and OS. However, the study sample size was small, and the independent prognostic value of the ApoB/ApoA-I was not explored. Furthermore, there were only 19 CRC patients with distant metastasis 20. Whether the ApoB/ApoA-I can serve as an independent prognostic factor of OS in mCRC patients remains unknown. We examined a large cohort of 838 mCRC patients to analyze the prognostic value of the pretreatment serum ApoB/ApoA-I on OS in mCRC patients. We also used the propensity score matching method to reduce the imbalance in the baseline characteristics to better identify the independent prognostic value of the ApoB/ApoA-I. Finally, we validated our main findings and conclusions with an independent cohort of 150 mCRC patients.

## Methods

### Patient selection and data collection

We retrospectively collected the clinical data of 1049 patients who were pathologically diagnosed with mCRC between Jan 2005 and Dec 2013 at Sun Yat-sen University Cancer Center (SYSUCC). The inclusion criteria of this study were as follows: 1) Eastern Cooperative Oncology Group performance status (ECOG PS) score ≤2, 2) received multidisciplinary treatment (MDT), and 3) available complete lab-based serum indexes before treatment. Exclusion criteria were as follows: 1) with previous or coexisting malignant tumors, 2) suffered from acute illnesses, including hepatic failure, severe trauma or stroke, 3) taking lipid-lowering drugs (statins, fibrates), antidiabetic drugs (oral antidiabetics, insulin) or corticosteroids, and 4) without complete follow-up data. We obtained the clinical data of participants, including patient demographics, clinicopathological information (tumor grade, primary tumor site, etc.) and clinical therapy (surgery, chemotherapy regimen, etc.) at first diagnosis from the patient electronic medical record (EMR) system. Serological biomarkers, including lipid metabolism indexes, carcinoembryonic antigen (CEA), and carbohydrate antigen 19-9 (CA19-9), were analyzed by a blood automatic biochemical analyzer. The patients were followed up every 3-6 months until death or dropout, with a median follow-up duration of 26 months (interquartile range (IQR): 15-45 months). A total of 838 patients (534 males, 304 females) were enrolled in our cohort. Then, we randomly divided 838 patients into two independent cohorts, namely, the training cohort (N=578) and the validation cohort (N=260).

An independent cohort of 150 mCRC patients hospitalized in SYSUCC between Jan 2014 and Dec 2014 was also included in this study to validate the independent prognostic value of ApoB/ApoA-I on OS in mCRC patients.

### Statistical analyses

According to clinical practice, we defined cholesterols >6.47 mmol/L, triglycerides > 1.70 mmol/L, HDL-C < 0.78 mmol/L, LDL-C >3.40 mmol/L, ApoA-I <1.05 ng/L, and ApoB >1.15 ng/L as dyslipidemia groups. We defined the best cut-off value of LHR at 2.55 and the ApoB/ApoA-I at 0.69 to stratify patients according to receiver operating characteristic (ROC) curve analysis in the training cohort. The cut-off values of LHR and the ApoB/ApoA-I were further validated in the validation cohort (N=260), the propensity score matching cohort (PSM) cohort (N=542) and the independent cohort (N=150). In this study, we explored the prognostic values of the serum lipid profiles of mCRC patients. A propensity score matching method was used to balance the baseline characteristics in the high and low ApoB/ApoA-I groups. The propensity scores of all patients were estimated by a logistic regression model using the following baseline characteristics as covariates: age, gender, ECOG PS score, primary tumor site, tumor grade, number of metastatic organs, surgery, chemotherapy, CEA and CA19-9. A one‐to‐one nearest neighbor matching algorithm with an optimal caliper of 0.2 without replacement was used to generate 542 patients, which were named the PSM cohort.

Differences in categorical variables or medians between groups were compared by the chi-square test and the Mann-Whitney test. Kaplan-Meier curves (log-rank test) and Cox proportional hazard regression models (backward method) were used to identify the independent prognostic factors of OS.

All statistical analyses were performed by using MedCalc (version 15.8; MedCalc Software bvba, Acacialaan, Belgium), SPSS (version 24.0; IBM Corp., Armonk, NY, USA) and SAS (version 9.1.3; SAS Institute, Inc., Cary, NC, USA). All statistical tests were two-sided, and P values less than 0.05 were considered statistically significant.

## Results

### Study flow chart and baseline characteristics

**Figure 1:**
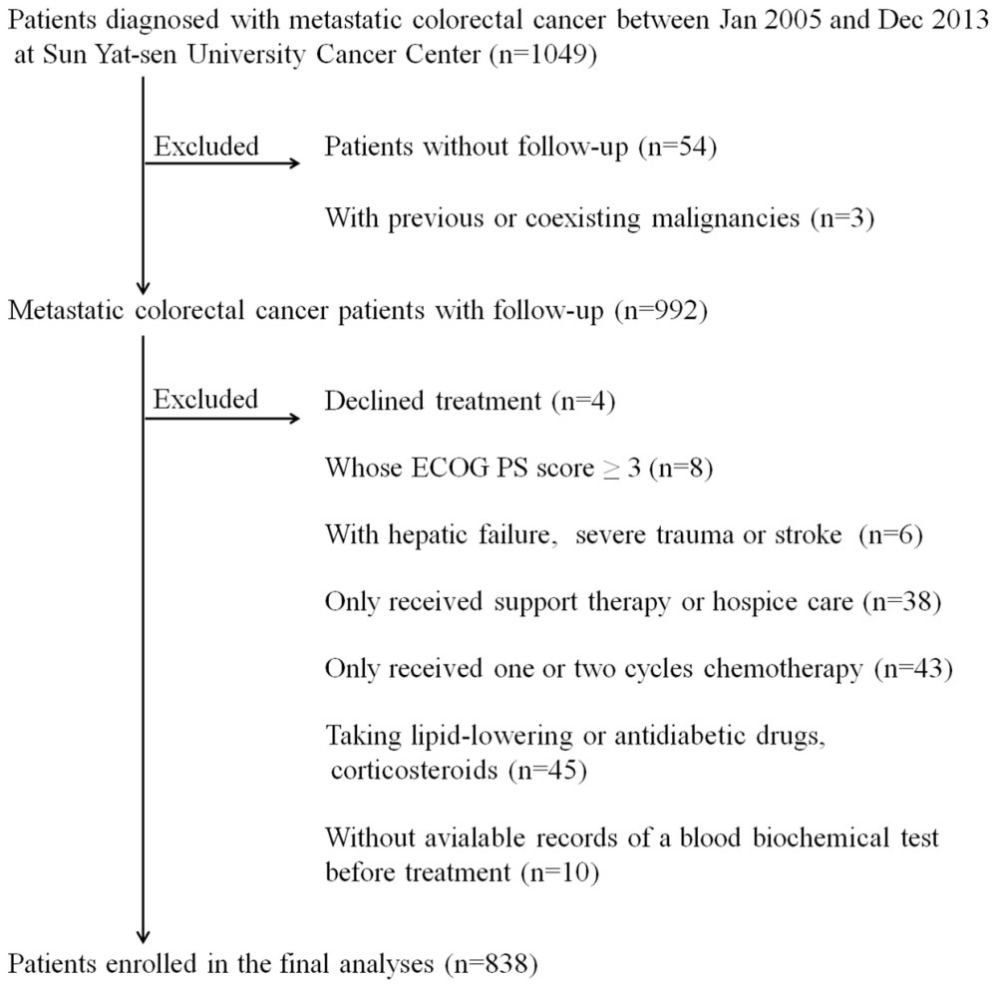
** The study flow chart.**^ *^ECOG PS score: Eastern Cooperative Oncology Group performance status score.

Figure 1 shows the details of the study flow chart. The clinical baseline characteristics of the training cohort (N=578) and the validation cohort (N=260) are described in table 1. We statistically compared the clinical parameters of these two cohorts and found that the two cohorts had comparable baseline characteristics. Patients in the two independent cohorts had similar age and gender distributions (training cohort: median age: 54 years, IQR: 43-62 years; gender: 64.4% males; validation cohort: median age: 55 years, IQR: 44-62 years; gender: 62.3% males). The primary tumor site was mostly located in the left semicolon (training cohort: 75.1%; validation cohort: 81.2%). The proportion of patients who suffered from multiple organ distant metastases was 59.0% and 56.5% in the training and validation cohort, respectively. Approximately half of the patients (training cohort: 46.9%; validation cohort: 44.2%) in both cohorts received radical or palliative surgery. The serum CEA and CA19-9 profiles were as follows: CEA >5 ng/ml: 67.3% and CA19-9 >35 ng/ml: 50.5% in the training cohort, CEA >5 ng/ml: 70.0% and CA19-9 >35 ng/ml: 54.2% in the validation cohort. As displayed in table 1, the number of patients whose serum ApoB/ApoA-I levels were under the cut-off value were 139 (24.0%) and 61 (23.5%) in the training and validation cohort, respectively.

The results shown in table 2 indicated that we successfully balanced the patients' baseline characteristics with the propensity score matching method, and 542 patients were included in the PSM cohort. The demographic and baseline characteristics of the PSM cohort (n=542) and the independent cohort (n=150) are shown in table 3. The median age of the PSM cohort was 53 years (IQR: 43-62 years), and most of the patients (63.8%) enrolled were male. The proportion of patients who received traditional chemotherapy combined with targeted therapy was 28%. In the PSM cohort, baseline variables, including age, gender, ECOG PS score, primary tumor site, tumor grade, number of metastatic organs, surgery, chemotherapy, CEA and CA19-9, were well balanced in the high ApoB/ApoA-I group and the low ApoB/ApoA-I group (table 2). Thus, the independent prognostic value of the ApoB/ApoA-I on OS in mCRC patients can be better explored.

### Survival Analysis

The endpoint of our research was OS, which was defined as the date from the initial diagnosis to death or the time of the last follow-up. The median OS for 838 patients was 22 months (IQR: 10.0-31.0).

**Figure 2:**
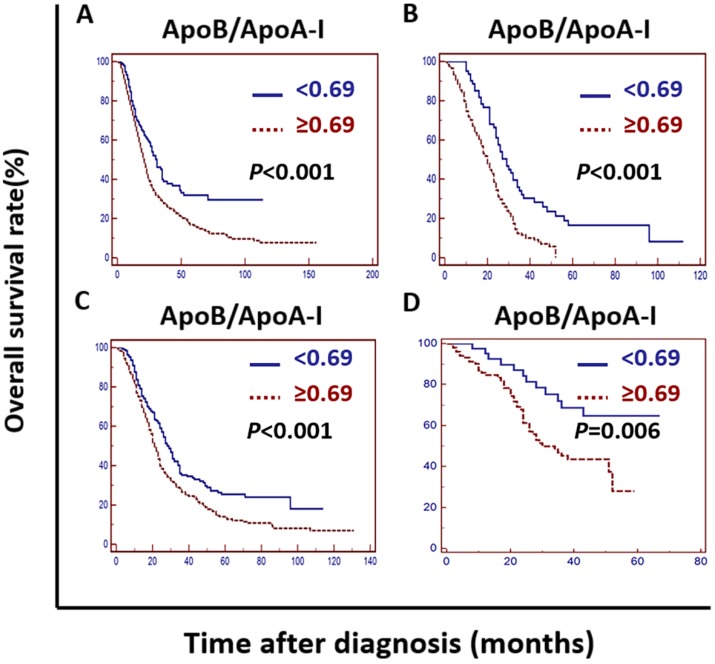
Kaplan-Meier curves depicting OS according to the ApoB/ApoA-I in the training cohort, the validation cohort, the PSM cohort and the independent cohort in patients with metastatic colorectal cancer. A: Kaplan-Meier analysis of OS in the training cohort. B: Kaplan-Meier analysis of OS in the validation cohort. C: Kaplan-Meier analysis of OS in the PSM cohort. D: Kaplan-Meier analysis of OS in the independent cohort.

Kaplan-Meier curve analysis indicated that an elevated ApoB/ApoA-I was associated with worse survival in the training cohort, the validation cohort, the PSM cohort and the independent cohort (*P* <0.01) (Figure 2).

### Univariate and multivariate analyses

**Figure 3:**
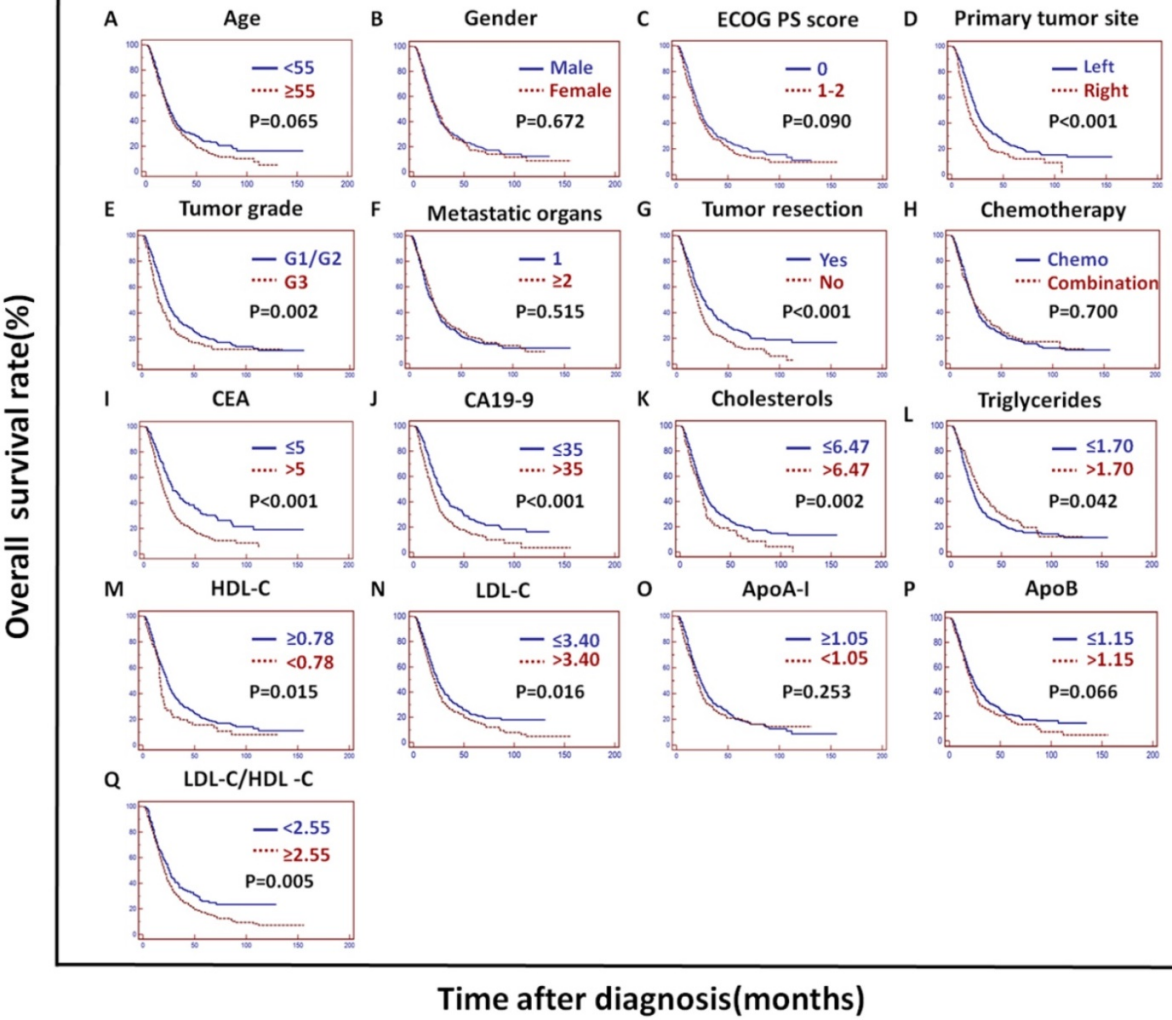
Kaplan-Meier curves depicting OS according to other factors in the training cohort. ^*^ECOG PS score: Eastern Cooperative Oncology Group performance status score; Metastatic organs: number of metastatic organs; Tumor resection: primary or metastatic tumor resection; CEA: serum carcinoembryonic antigen; CA19-9: carbohydrate antigen 19-9; HDL-C: high-density lipoprotein cholesterol; LDL-C: low-density lipoprotein cholesterol; ApoA-I: apolipoprotein A-I; ApoB: apolipoprotein B; LDL-C/HDL-C: LDL-C to HDL-C ratio.

In the training cohort, univariate analysis showed that the primary tumor site, tumor grade, tumor resection, CEA, CA19-9, cholesterols, triglycerides, HDL-C, LDL-C, LDL-C/HDL-C and ApoB/ApoA-I were prognostic of OS (Figures 2A and 3).

**Figure 4:**
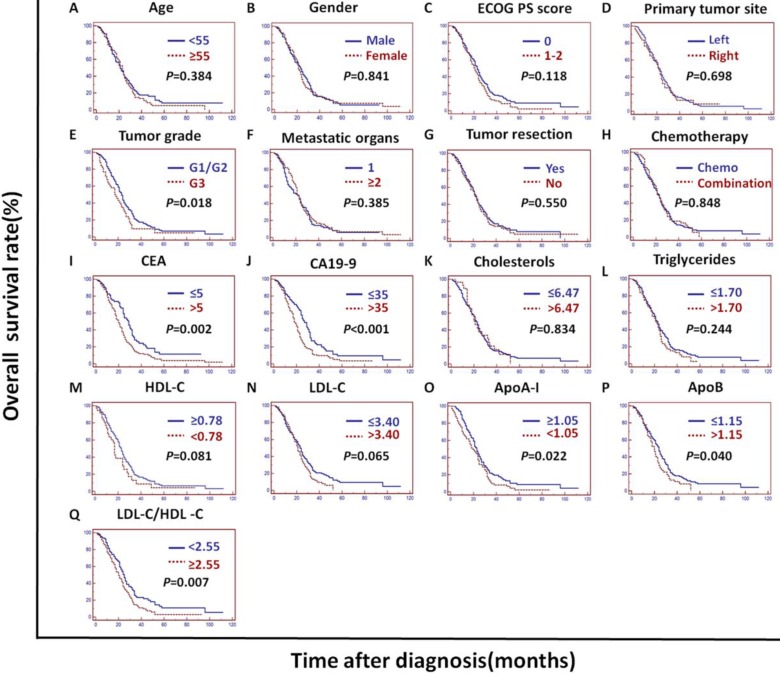
Kaplan-Meier curves depicting OS according to other factors in the validation cohort.^ *^ECOG PS score: Eastern Cooperative Oncology Group performance status score; Metastatic organs: number of metastatic organs; Tumor resection: primary or metastatic tumor resection; CEA: serum carcinoembryonic antigen; CA19-9:; carbohydrate antigen 19-9; HDL-C: high-density lipoprotein cholesterol; LDL-C: low-density lipoprotein cholesterol; ApoA-I: apolipoprotein A-I; ApoB: apolipoprotein B; LDL-C/HDL-C: LDL-C to HDL-C ratio.

In the validation cohort, univariate analysis showed that the tumor grade, CEA, CA19-9, ApoA-I, ApoB, LDL-C/HDL-C and ApoB/ApoA-I were prognostic of OS (Figures 2B and 4).

**Figure 5:**
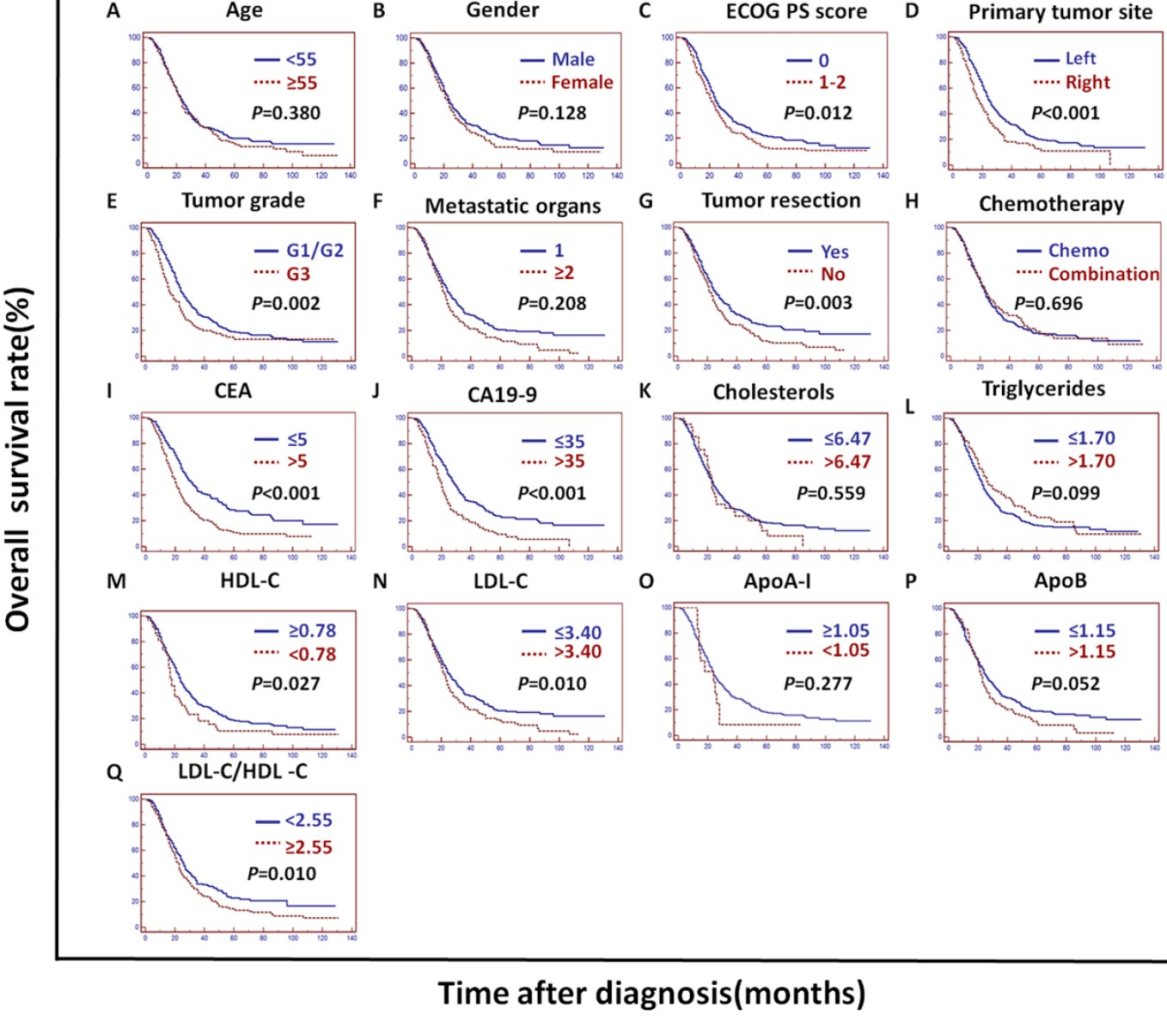
Kaplan-Meier curves depicting OS according to other factors in the PSM cohort. ^*^PSM: propensity score matching; ECOG PS score: Eastern Cooperative Oncology Group performance status score; Metastatic organs: number of metastatic organs; Tumor resection: primary or metastatic tumor resection; CEA: serum carcinoembryonic antigen; CA19-9: carbohydrate antigen 19-9; HDL-C: high-density lipoprotein cholesterol; LDL-C: low-density lipoprotein cholesterol; ApoA-I: apolipoprotein A-I; ApoB: apolipoprotein B; LDL-C/HDL-C: LDL-C to HDL-C ratio.

In the PSM cohort, univariate analysis showed that the ECOG PS score, primary tumor site, tumor grade, tumor resection, CEA, CA19-9, HDL-C, LDL-C, LDL-C/HDL-C and ApoB/ApoA-I were prognostic of OS (Figures 2C and 5).

The tumor grade, CEA, CA19-9, LDL-C/HDL-C and ApoB/ApoA-I were prognostic of OS in all three cohorts (Figures 3, 4, and 5). Variables significantly prognostic of OS identified by univariate analysis were further analyzed by Cox proportional hazards regression models (backward method) in multivariate analysis. The ApoB/ApoA-I was identified as an independent prognostic factor for OS in all three cohorts [training cohort: hazard ratio (HR): 1.371; 95% confidence interval (CI): 1.205-1.870, *P*=0.045; validation cohort: HR: 1.924 95% CI: 1.360-2.723, *P*<0.001; PSM cohort: HR: 1.599 95% CI: 1.287-1.988, *P*<0.001, tables 4, 5, and 6].

CEA, CA19-9 and tumor grade were also identified as independent prognostic factors for OS in all three cohorts. The LDL-C/HDL-C was identified as an independent prognostic factor for OS in the training cohort. However, the LDL-C/HDL-C was not identified as an independent prognostic factor for OS in the validation cohort or the PSM cohort (Tables 4, 5, and 6).

Univariate and multivariate analyses showed that ApoB/ApoA-I was an independent prognostic factor in the independent cohort (HR: 1.949; 95% CI: 1.014-3.747, P=0.046) (Tables 7).We validated our main findings and conclusions with an independent cohort.

## Discussion

We confirmed that an increased ApoB/ApoA-I was an independent prognostic factor for shorter OS in mCRC patients by exploring the prognostic values of clinicopathological variables in the training cohort, the validation cohort, the PSM cohort and the independent cohort. In our research, we explored the independent prognostic value of the ApoB/ApoA-I using the traditional randomization method to generate the training cohort and the validation cohort and using the propensity matching scoring method to generate the PSM cohort. Finally, we validated our main findings and conclusions with an independent cohort.

To the best of our knowledge, we identified for the first time the independent prognostic value of the ApoB/ApoA-I in a large cohort of mCRC patients.

ApoA-I is an important apolipoprotein that reversely transports cholesterols from peripheral tissues to the liver. It has been reported to be a cofactor for lectin cholesterol acyltransferase (LCAT), which extensively participates in lipid metabolism^24^. In recent decades, many studies have reported the effect of lipid metabolism indexes on cancers, most of which focused on the risk of cancer influenced by lipids or lipoproteins. Several studies have identified that high ApoA-I can sharply increase the risk of breast cancer but inversely reduce the risk of lung cancer 4, 5, 7, 25-28. The prognostic value of serum ApoA-I has been previously investigated, and the results of different studies confirmed that increased ApoA-I is a favorable factor in ovarian cancer, lung cancer, kidney cancer, nonmetastatic nasopharyngeal cancer and mCRC for OS. A recent retrospective study reported that low serum ApoA-I levels were associated with advanced T class and TNM stage and systemic inflammation biomarkers in CRC. All of these findings indicate that serum ApoA-I may participate in multiple processes in the occurrence and development of cancers 19, 20, 29-31.

On the other hand, ApoB is known as a carrier that ships lipids, including cholesterols and triglycerides, into extrahepatic tissues. In a large-scale prospective cohort study, the investigators found that high circulating ApoB could increase the risk of CRC in men but reduce the risk of breast cancer in women. Furthermore, elevated blood ApoB can result in increased prevalence rates of CRC and lung cancer 4, 5, 32. A high ApoB/ApoA-I was defined as a risk factor for atherosclerosis, hypertension and polycystic ovary syndrome (PCOS) 33. Only one study confirmed that a low ApoB/ApoA-I were favorable prognostic factors in CRC. The conclusion of this research was based on a small sample size, and most of the patients were diagnosed with early colorectal carcinoma. Only 19 CRC patients had distant metastasis 20. The independent prognostic value of the ApoB/ApoA-I in mCRC remains unknown. Therefore, we examined a large cohort of 838 patients and confirmed the independent prognostic value of the ApoB/ApoA-I in mCRC patients.

Numerous studies have indicated that ApoA-I and ApoB play a non-negligible role in malignancies, but the underlying mechanisms are not fully known. Accumulating investigations revealed the potential mechanisms of these phenomena as follows: 1) According to several studies, ApoA-I is negatively associated with tumor-induced systemic inflammation, while elevated ApoB indicates a higher systemic inflammatory marker. Furthermore, a high ApoB/ApoA-I, which is considered atherogenic, may contribute to tumor necrosis^34^, ^35^; 2) A research group found that ApoA-I and its mimetic peptides can reduce the viability and proliferation of ovarian carcinoma cells *in vivo* and *in vitro*^36^; 3) Additionally, some studies indicated that apolipoprotein can initiate cellular signal transduction and promote antitumor drug delivery^14^, ^37^, ^38^; 4) In several studies, ApoA-I potently suppressed tumor growth and metastasis by both innate and adaptive immune processes^12^, ^13^, ^39^, ^40^.

The advantages of our study are described below. First, our study enrolled a large cohort of 838 CRC patients, and all the patients were diagnosed with mCRC. Second, we randomly assigned the patients into the training cohort and the validation cohort. To avoid a potential statistical bias, we also identified the independent prognostic value in a PSM cohort. The independent prognostic value of the ApoB/ApoA-I on OS in mCRC was confirmed in all three cohorts. We also validated our main findings and conclusions with an independent cohort. Third, we explored the prognostic value of the ApoB/ApoA-I and the LDL-C to HDL-C ratio (LHR) at the same time. The ApoB/ApoA-I was identified as an independent prognostic factor in all three cohorts, while LHR was identified as an independent prognostic factor in the training cohort only. However, there were several limitations to this study. First, all the patients were from a single cancer center, and all the patients were Chinese. The conclusion may not be suitable for Western populations. Second, the exact mechanism by which the ApoB/ApoA-I affects prognosis remains unknown. Third, although we used propensity score matching to reduce the imbalance in the baseline clinical variables, a selection bias might not have been completely avoided because of the retrospective nature of this research.

## Conclusion

In conclusion, the serum ApoB/ApoA-I is an independent prognostic factor for OS in mCRC patients. The prognostic value of the ApoB/ApoA-I should be further validated in a larger prospective and multicenter study.

## Figures and Tables

**Table 1:** Baseline demographics and clinical characteristics of the training cohort and the validation cohort.

Characteristics	Training Cohort, n (%)	Validation Cohort, n (%)	*P* value
Total	578(100.0)	260(100.0)	
Age, years (median/IQR)	54 (43-62)	55(44-62)	0.805
Gender			0.587
Male	372 (64.4)	162(62.3)	
Female	206(35.6)	98(37.7)	
ECOG PS score			0.879
0	343(59.3)	162(62.3)	
1-2	235(40.7)	98(37.7)	
Primary tumor site			0.062
Right	144(24.9)	49(18.8)	
Left	434(75.1)	211(81.2)	
Tumor grade			0.440
G1-2	427(73.9)	199(76.5)	
G3	151(26.1)	61(23.5)	
Number of metastatic organs			0.545
1	237 (41.0)	113(43.5)	
≥2	341(59.0)	147(56.5)	
Primary/Metastatic tumor resection			0.501
No	307(53.1)	145(55.8)	
Yes	271 (46.9)	115(44.2)	
Chemotherapy			0.281
Chemo	410(70.9)	194(74.6)	
Combination	168(29.1)	66(25.4)	
CEA (ng/ml)			0.471
>5	389(67.3)	182(70.0)	
≤5	189(32.7)	78(30.0)	
CA19-9 (ng/ml)			0.332
>35	292(50.5)	141(54.2)	
≤35	286(49.5)	119(45.8)	
Cholesterols (mmol/L)			0.557
>6.47	63(10.9)	32(12.3)	
≤6.47	515(89.1)	228(87.7)	
Triglycerides (mmol/L)			0.793
>1.70	139(24.0)	60(23.1)	
≤1.70	439(76.0)	200(76.9)	
HDL-C (mmol/L)			0.664
≥0.78	504(87.2)	224(86.2)	
< 0.78	74(12.8)	36(13.8)	
LDL-C (mmol/L)			0.365
>3.40	236(40.8)	115(44.2)	
≤3.40	342(59.2)	145(55.8)	
ApoA-I (ng/L)			0.938
≥1.05	371(64.2)	166(63.8)	
<1.05	207(35.8)	94(36.2)	
ApoB (ng/L)			0.288
>1.15	165(28.5)	84(32.3)	
≤1.15	413(71.5)	176(67.7)	
LDL-C/HDL-C			0.877
≥2.55	366(63.3)	97(37.3)	
<2.55	212(36.7)	163(62.7)	
ApoB/ApoA-I			0.930
≥0.69	439(76.0)	199(76.5)	
<0.69	139(24.0)	61(23.5)	

IQR: interquartile range; ECOG PS score: Eastern Cooperative Oncology Group performance status score; CEA: serum carcinoembryonic antigen; CA19-9: carbohydrate antigen 19-9; HDL-C: high-density lipoprotein cholesterol; LDL-C: low-density lipoprotein cholesterol; ApoA-I: apolipoprotein A-I; ApoB: apolipoprotein B; LDL-C/HDL-C: LDL-C to HDL-C ratio; ApoB/ApoA-I: ApoB to ApoA-I ratio.

**Table 2:** Association analysis of colorectal cancer patients before propensity score matching (n=838) and after propensity score matching (n=542).

Cohort	CRC patients before PSM (n=838)			CRC patients after PSM (n=542)		
Characteristics	ApoB/ApoA-I Low, n (%)	ApoB/ApoA-I High, n (%)	*P*-value	ApoB/ApoA-I Low, n (%)	ApoB/ApoA-I High, n (%)	*P*-value
Total	200(100%)	638(100%)		191(100%)	351(100%)	
Age, years			0.030			0.603
<55	116(58.0)	314(49.2)		110(57.6)	194(55.3)	
≥55	84(42.0)	324(50.8)		81(42.4)	157(44.7)	
Gender			0.111			0.095
Male	118(59.0)	416(65.2)		113(59.2)	233(66.4)	
Female	82(41.0)	222(34.8)		78(40.8)	118(33.6)	
ECOG PS score			0.365			0.412
0	126(63.0)	379(59.4)		119(62.3)	206(58.7)	
1-2	74(37.0)	259(40.6)		72(37.7)	145(41.3)	
Primary tumor site			< 0.001			0.110
Right	65(32.5)	128(20.1)		72(37.7)	81(23.1)	
Left	135(67.5)	510(79.9)		119(62.3)	270(76.9)	
Tumor grade			0.911			0.634
G1-G2	150(75.0)	476(74.6)		144(75.4)	271(77.2)	
G3	50(25.0)	162(25.4)		47(24.6)	80(22.8)	
Number of metastatic organs			0.569			0.349
1	87(43.5)	263(41.2)		83(43.5)	138(39.3)	
≥2	113(56.5)	375(58.8)		108(56.5)	213(60.7)	
Primary/Metastatic tumor resection			0.599			0.599
No	109(54.5)	343(53.8)		103(53.9)	181(51.6)	
Yes	91(45.5)	295(46.2)		88(46.1)	170(48.4)	
Chemotherapy			0.216			0.265
Chemo	151(75.5)	453(71.0)		143(74.9)	247(70.4)	
Combination	49(24.5)	185(29.0)		48(25.1)	104(29.6)	
CEA (ng/ml)			0.013			0.736
>5	122(61.0)	449(70.4)		118(61.8)	222(63.2)	
≤5	78(39.0)	189(29.6)		73(38.2)	129(36.8)	
CA19-9 (ng/ml)			< 0.001			0.610
>35	82(41.0)	351(55.0)		80(41.9)	155(44.2)	
≤35	118(59.0)	287(45.0)		111(58.1)	196(55.8)	

^*^ PSM: propensity score matching; ECOG PS score: Eastern Cooperative Oncology Group Performance Status score; CEA: serum carcinoembryonic antigen; CA19-9: carbohydrate antigen 19-9.

**Table 3:** Baseline demographics and clinical characteristics of the propensity score matching (PSM) cohort (n=542) and the independent cohort (n=150).

Characteristics	PSM cohort, n (%)	Independent cohort, n (%)
Total	542(100.0)	150(100.0)
Age, years (median/IQR)	53(43-62)	61(51-67)
Gender		
Male	346(63.8)	88 (58.7)
Female	196(36.2)	62(41.3)
ECOG PS score		
0	325(60.0)	22(14.7)
1-2	217(40.0)	128(85.3)
Primary tumor site		
Right	137(25.3)	32(21.3)
Left	405(74.7)	118(78.7)
Tumor grade		
G1-2	415(76.6)	112(74.7)
G3	127(23.4)	38(25.3)
Number of metastatic organs		
1	221(40.8)	102(68.0)
≥2	321(59.2)	48(32.0)
Primary/Metastatic tumor resection		
No	284(52.4)	43(28.7)
Yes	258(47.6)	107(71.3)
Chemotherapy		
Chemo alone	390(72.0)	104(69.3)
Combination	152(28.0)	46(30.7)
CEA (ng/ml)		
>5	340(62.7)	105(70.0)
≤5	202(37.3)	45(30.0)
CA19-9(ng/ml)		
>35	235(43.4)	67(44.7)
≤35	307(56.6)	83(55.3)
Cholesterols (mmol/L)		
>6.47	45(8.3)	18(12.0)
≤6.47	497(91.7)	132(88.0)
Triglycerides (mmol/L)		
>1.70	116(21.4)	24(16.0)
≤1.70	426(78.6)	126(84.0)
HDL-C (mmol/L)		
≥0.78	481(88.7)	140(93.3)
<0.78	61(11.3)	10(6.7)
LDL-C (mmol/L)		
>3.40	186(34.3)	53(35.3)
≤3.40	356(65.7)	97(64.7)
ApoA-I (ng/L)		
≥1.05	362(66.8)	49(32.7)
<1.05	180(33.2)	101(67.3)
ApoB (ng/L)		
>1.15	129(23.8)	40(26.7)
≤1.15	413(76.2)	110(73.3)
LDL-C/HDL-C		
≥2.55	297(54.8)	92(61.3)
<2.55	245(45.2)	58(38.7)
ApoB/ApoA-I		
≥0.69	351(64.8)	106(70.7)
<0.69	191(35.2)	44(29.3)

^*^IQR: interquartile range; ECOG PS score: Eastern Cooperative Oncology Group performance status score; CEA: serum carcinoembryonic antigen; CA19-9: carbohydrate antigen 19-9; HDL-C: high-density lipoprotein cholesterol; LDL-C: low-density lipoprotein cholesterol; ApoA-I: apolipoprotein A-I; ApoB: apolipoprotein B; LDL-C/HDL-C: LDL-C to HDL-C ratio; ApoB/ApoA-I: ApoB to ApoA-I ratio.

**Table 4:** Univariate and multivariate analyses of the prognostic factors of overall survival (OS) in the training cohort.

Variables	Univariate			Multivariate		
	HR	95% CI	*P* value	HR	95% CI	*P-* value
Age, years (<55/≥55)	1.198	0.986-1.456	0.070			
Gender (Male/Female)	1.044	0.853-1.277	0.676			
ECOG PS score (0/1-2)	1.184	0.971-1.443	0.095			
Primary tumor site ( Left /Right)	1.477	1.185-1.841	0.001	1.437	1.175-1.757	< 0.001
Tumor grade (G1-2/G3)	1.410	1.128-1.762	0.003	1.565	1.245-1.966	< 0.001
Number of metastatic organs (1/≥2)	0.937	0.768-1.143	0.521			
Primary/Metastatic tumor resection (Yes/No)	1.443	1.185-1.758	0.001	1.473	1.175-1.757	< 0.001
Chemotherapy (Chemo/combination)	0.960	0.776-1.187	0.705			
CEA (ng/ml) (<5/≥5)	1.692	1.364-2.100	< 0.001	1.501	1.193-1.889	0.001
CA19-9 (ng/ml) (<35/≥35)	1.569	1.289-1.911	< 0.001	1.317	1.065-1.630	0.011
Cholesterols (mmol/L) (≤6.47/>6.47)	1.375	1.033-1.8230	0.006			
Triglycerides (mmol/L) (≤1.70/>1.70)	0.789	0.625-0.996	0.046	0.707	0.558-0.896	0.004
HDL-C (mmol/L) (≥0.78/<0.78)	1.415	1.064-1.882	0.017			
LDL-C (mmol/L) (≤3.40/>3.40)	1.266	1.041-1.540	0.018			
ApoA-I(ng/L) (≥1.05/<1.05)	1.124	0.917-1.378	0.260			
ApoB (ng/L) (≤1.15/>1.15)	1.123	0.984-1.496	0.071			
LDL-C/HDL-C (<2.55/≥2.55)	1.340	1.087-1.651	0.006	1.334	1.109-1.746	0.036
ApoB/ApoA-I (<0.69/≥0.69)	1.580	1.234-2.023	< 0.001	1.371	1.205-1.870	0.045

^*^ ECOG PS score: Eastern Cooperative Oncology Group performance status score; CEA: serum carcinoembryonic antigen; CA19-9: carbohydrate antigen 19-9; HDL-C: high-density lipoprotein cholesterol; LDL-C: low-density lipoprotein cholesterol; ApoA-I: apolipoprotein A-I; ApoB: apolipoprotein B; LDL-C/HDL-C: LDL-C to HDL-C ratio; ApoB/ApoA-I: ApoB to ApoA-I ratio.

**Table 5:** Univariate and multivariate analyses of the prognostic factors of overall survival (OS) in the validation cohort.

Variables	Univariate			Multivariate		
	HR	95% CI	*P* value	HR	95% CI	*P-* value
Age, years (<55/≥55)	1.029	0.774-1.368	0.388			
Gender (Male/Female)	1.044	0.853-1.277	0.876			
ECOG PS score (0/1-2)	1.248	0.939-1.658	0.127			
Primary tumor site ( Left /Right )	1.072	0.749-1.533	0.705			
Tumor grade (G1-2/G3)	1.475	1.059-2.056	0.022	1.635	1.163-2.300	0.005
Number of metastatic organs (1/≥2)	0.886	0.669-1.173	0.397			
Primary/Metastatic tumor resection (Yes/No)	1.087	0.822-1.438	0.559			
Chemotherapy (Chemo/combination)	0.970	0.706-1.333	0.852			
CEA (ng/ml) (<5/≥5)	1.605	1.170-2.203	0.003	1.441	1.029-2.016	0.033
CA19-9 (ng/ml) (<35/≥35)	1.664	1.251-2.214	< 0.001	1.489	1.101-2.015	0.010
Cholesterols (mmol/L) (≤6.47/>6.47)	0.955	0.618-1.477	0.855			
Triglycerides (mmol/L) (≤1.70/>1.70)	1.208	0.872-1.673	0.256			
HDL-C (mmol/L) (≥0.78/<0.78)	1.424	0.947-2.140	0.089			
LDL-C (mmol/L) (≤3.40/>3.40)	1.298	0.996-1.726	0.072			
ApoA-I (ng/L) (≥1.05/<1.05)	1.391	1.040-1.863	0.026			
ApoB (ng/L) (≤1.15/>1.15)	1.356	1.005-1.861	0.046			
LDL-C/HDL-C (<2.55/≥2.55)	1.491	1.107-2.009	0.008			
ApoB/ApoA-I (<0.69/≥0.69)	2.122	1.504-2.995	< 0.001	1.924	1.360-2.723	<0.001

*ECOG PS score: Eastern Cooperative Oncology Group performance status score; CEA: serum carcinoembryonic antigen; CA19-9: carbohydrate antigen 19-9; HDL-C: high-density lipoprotein cholesterol; LDL-C: low-density lipoprotein cholesterol; ApoA-I: apolipoprotein A-I; ApoB: apolipoprotein B; LDL-C/HDL-C: LDL-C to HDL-C ratio; ApoB/ApoA-I: ApoB to ApoA-I ratio.

**Table 6:** Univariate and multivariate analyses of the prognostic factors of overall survival (OS) in the PSM cohort.

Variables	Univariate			Multivariate		
	HR	95% CI	*P* value	HR	95% CI	*P-* value
Age, years (<55/≥55)	1.093	0.893-1.399	0.387			
Gender (Male/Female)	1.171	0.952-1.440	0.134			
ECOG PS score (0/1-2)	1.295	1.056-1.588	0.013	1.268	1.032-1.557	0.024
Primary tumor site ( Left /Right )	1.531	1.207-1.897	<0.001	1.491	1.187-1.873	0.001
Tumor grade (G1-2/G3)	1.445	1.141-1.831	0.003	1.448	1.141-1.836	0.002
Number of metastatic organs (1/≥2)	0.878	0.716-1.078	0.216			
Primary/Metastatic tumor resection (Yes/No)	1.347	1.100-1.650	0.004	1.309	1.066-1.606	0.010
Chemotherapy (Chemo/combination)	0.957	0.767-1.195	0.700			
CEA (ng/ml) (<5/≥5)	1.751	1.410-2.173	<0.001	1.573	1.249-1.981	<0.001
CA19-9 (ng/ml) (<35/≥35)	1.721	1.401-2.115	<0.001	1.469	1.181-1.828	0.001
Cholesterols (mmol/L) (≤6.47/>6.47)	1.109	0.779-1.578	0.565			
Triglycerides (mmol/L) (≤1.70/>1.70)	0.814	0.634-1.044	0.105			
HDL-C (mmol/L) (≥0.78/<0.78)	1.413	1.034-1.932	0.030			
LDL-C (mmol/L) (≤3.40/>3.40)	1.308	1.062-1.612	0.012			
ApoA-I (ng/L) (≥1.05/<1.05)	1.386	0.760-2.527	0.287			
ApoB (ng/L) (≤1.15/>1.15)	1.250	0.993-1.574	0.057			
LDL-C/HDL-C (<2.55/≥2.55)	1.299	1.059-1.594	0.012			
ApoB/ApoA-I (<0.69/≥0.69)	1.485	1.196-1.843	< 0.001	1.599	1.287-1.988	<0.001

^*^ PSM cohort: propensity score matching cohort; ECOG PS score: Eastern Cooperative Oncology Group performance status score; CEA: serum carcinoembryonic antigen; CA19-9: carbohydrate antigen 19-9; HDL-C: high-density lipoprotein cholesterol; LDL-C: low-density lipoprotein cholesterol; ApoA-I: apolipoprotein A-I; ApoB: apolipoprotein B; LDL-C/HDL-C: LDL-C to HDL-C ratio; ApoB/ApoA-I: ApoB to ApoA-I ratio.

**Table 7:** Univariate and multivariate analyses of the prognostic factors of overall survival (OS) in the independent cohort.

Variables	Univariate			Multivariate			
	HR	95% CI	*P* value	HR	95% CI	*P-* value	
Age, years (<55/≥55)	1.099	0.627-1.925	0.744				
Gender (Male/Female)	0.870	0.516-1.466	0.603				
ECOG PS score (0/1-2)	0.594	0.308-1.144	0.121				
Primary tumor site ( Left /Right )	0.765	0.406-1.440	0.408				
Tumor grade (G1-2/G3)	0.986	0.557-1.746	0.961				
Number of metastatic organs (1/≥2)	1.467	0.862-2.499	0.160				
Primary/Metastatic tumor resection (Yes/No)	1.970	1.154-3.360	0.013	1.652	0.960-2.843	0.071	
Chemotherapy (Chemo/combination)	1.138	0.673-1.925	0.631				
CEA (ng/ml) (<5/≥5)	2.474	1.327-4.611	0.005	1.980	1.041-3.766	0.038	
CA19-9 (ng/ml) (<35/≥35)	1.826	1.095-3.043	0.022				
Cholesterols (mmol/L) (≤6.47/>6.47)	2.141	1.038-4.417	0.040				
Triglycerides (mmol/L) (≤1.70/>1.70)	0.932	0.460-1.888	0.845				
HDL-C (mmol/L) (≥0.78/<0.78)	1.529	0.614-3.806	0.365				
LDL-C (mmol/L) (≤3.40/>3.40)	1.215	0.709-2.081	0.480				
ApoA-I (ng/L) (≥1.05/<1.05)	1.284	0.745-2.213	0.370				
ApoB (ng/L) (≤1.15/>1.15)	1.535	0.874-2.697	0.138				
LDL-C/HDL-C (<2.55/≥2.55)	1.309	0.774-2.215	0.317				
ApoB/ApoA-I (<0.69/≥0.69)	2.391	1.264-4.524	0.008	1.949	1.014-3.747	0.046	

^*^ ECOG PS score: Eastern Cooperative Oncology Group performance status score; CEA: serum carcinoembryonic antigen; CA19-9: carbohydrate antigen 19-9; HDL-C: high-density lipoprotein cholesterol; LDL-C: low-density lipoprotein cholesterol; ApoA-I: apolipoprotein A-I; ApoB: apolipoprotein B; LDL-C/HDL-C: LDL-C to HDL-C ratio; ApoB/ApoA-I: ApoB to ApoA-I ratio.

## Abbreviations

ApoA-I: apolipoprotein A-I
ApoB: apolipoprotein B
ApoB to ApoA-I ratio: ApoB/ApoA-I
mCRC: metastatic colorectal cancer
ROC: receiver operating characteristic
PSM: propensity score matching
OS: overall survival
HR: hazard ratio
CI: confidence interval
MDT: multidisciplinary treatment
EMR: electronic medical record
IQR: interquartile range
ECOG PS score: Eastern Cooperative Oncology Group performance status score
CEA: serum carcinoembryonic antigen
CA19-9: carbohydrate antigen 19-9
HDL-C: high-density lipoprotein cholesterol
LDL-C: low-density lipoprotein cholesterol
LDL-C/HDL-C: LDL-C to HDL-C ratio
